# Structural Control of Nanofibers According to Electrospinning Process Conditions and Their Applications

**DOI:** 10.3390/mi14112022

**Published:** 2023-10-30

**Authors:** Trong Danh Nguyen, Sojeong Roh, My Thi Ngoc Nguyen, Jun Seop Lee

**Affiliations:** Department of Materials Science and Engineering, Gachon University, 1342 Seongnam-Daero, Sujeong-Gu, Seongnam-Si 13120, Gyeonggi-Do, Republic of Korea; ntdanh041@gachon.ac.kr (T.D.N.); hihithwjd@gachon.ac.kr (S.R.); ngocmy@gachon.ac.kr (M.T.N.N.)

**Keywords:** electrospinning, nanofibers, carbon fibers, polymer fibers, composite fibers

## Abstract

Nanofibers have gained much attention because of the large surface area they can provide. Thus, many fabrication methods that produce nanofiber materials have been proposed. Electrospinning is a spinning technique that can use an electric field to continuously and uniformly generate polymer and composite nanofibers. The structure of the electrospinning system can be modified, thus making changes to the structure, and also the alignment of nanofibers. Moreover, the nanofibers can also be treated, modifying the nanofiber structure. This paper thoroughly reviews the efforts to change the configuration of the electrospinning system and the effects of these configurations on the nanofibers. Excellent works in different fields of application that use electrospun nanofibers are also introduced. The studied materials functioned effectively in their application, thereby proving the potential for the future development of electrospinning nanofiber materials.

## 1. Introduction

Nanofiber materials have attracted much attention because of their extraordinary advantages that can bring many benefits to applications [1]. Since the interaction of the materials with the working environment happens on their surface, improvement of the surface area can greatly enhance their working performance. Many studies have proposed that nanofiber materials possess a surface area much greater than bulk or 2D (sheet) materials [2,3]. Meanwhile, they are still imbued with the physical properties of the material from which they are built. These properties can help the materials to be applied in some applications that require a certain physical strength [4,5,6,7,8].

Many fabrication methods for nanofibers have been proposed in the literature, including the uses of hard, soft, and free templates. While hard and soft templates release a large amount of chemicals into the environment, the free template seems to be more environment-friendly, in which case, electrospinning is a free template technique that uses an electrical field to fabricate the polymer and polymer composite nanofibers [9,10,11,12,13]. This technique requires a very limited amount of solvent, which is used to dissolve the polymer into a high-viscosity solution. After the shape of fibers is applied to the solution, the solvent is released and leaves the material in the shape of the nanofibers [14]. The polymer used in the process must be dissolved in the solvent, which limits the selection of polymers that can be spun. However, some of the available commercial polymers are dissolvable in a low boiling point solvent, so such limitation is almost negligible.

Though the mechanism of electrospinning is simple, many improvements can be carried out on the components of the system [15,16,17]. Thereby, the morphology of the produced nanofibers can be greatly altered. There are reports that described further treatment processes that have been carried out on the electrospun nanofibers [18]. As collected, the nanofibers usually stack over each other, layer after layer, and form a membrane. They can be used as a membrane sheet and built up to a 3D structure [19,20,21]. On the other hand, carbonization can transform most of the existing polymer nanofibers into carbon nanofibers (CNFs). These CNFs showed great potential in electrochemical application, which has gained increased interest in recent years [22].

Herein, the manuscript proposes a critical review of the electrospinning system. First, the modifications made to components (electrospun material, nozzle, and collector) of the electrospinning system are introduced, together with the effects on the shape of nanofibers. Later, the recent applications of nanofibers that are fabricated by electrospinning are suggested. Many modifications of nanofibers, including freeze-drying, deposition, and thermal treatment, can be applied to achieve suitable properties for many advanced applications.

## 2. The Electrospinning System

**Figure 1:**
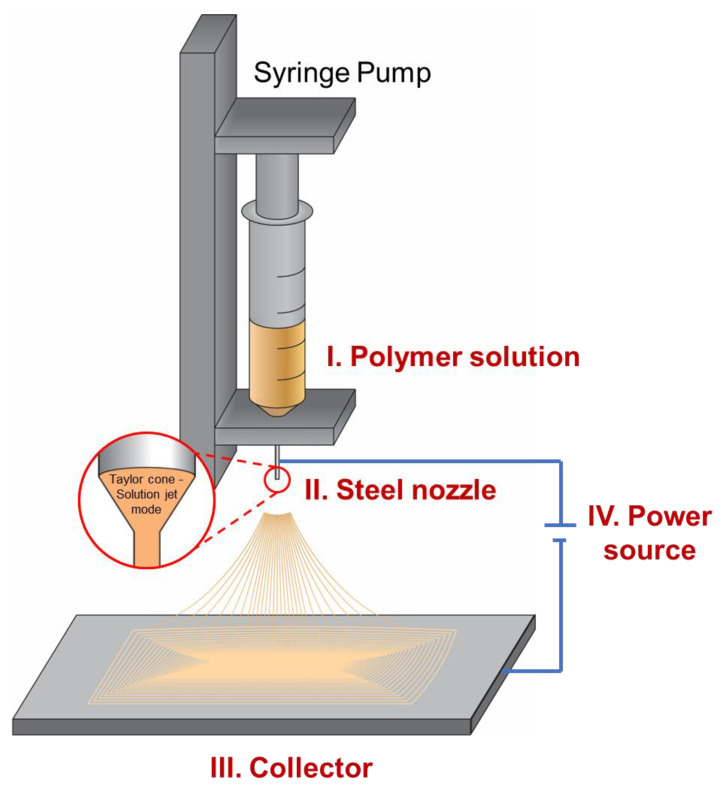
Standard electrospinning system, including a syringe pump, a syringe containing electrospun solution, a steel nozzle, a planar collector, and a power source.

The basic electrospinning system consists of a syringe pump, material solution, nozzle, collector, and power source (Figure 1). The materials used in an electrospinning system are usually solutions where the polymers are well dissolved in an easy-to-vaporize solvent. The syringe pump injects the polymer solution from a syringe into the connected metal nozzle, where it is charged by a high-voltage electric field. The polymer solution inherits the charge from the nozzle, which is opposite to the charge of the collector [23]. Thus, the solutions are drawn toward the surface of the collector. Here, two processes happen simultaneously. First, while the droplet of the polymer solution is held at the outlet of the nozzle, it is stretched and pulled over the collector [24,25]. During this process, the high difference between the areas of the nozzle and the collector splits the solutions and forms nanoscale diameter fibers [26]. Secondly, the split solution has a high surface area, which makes it easier for the solvent trapped between the polymer matrix to be released, leaving solid polymer fibers. Despite the simple mechanism and advantages in product quantity, many factors can affect the outcome properties of the fibers and need to be optimized thoroughly with each material and condition. These factors may derive from the materials and systems configuration, as well as the environment [27]. The necessary power needed to operate an electrospinning system is very high. There is no electron flow (amplitude is near zero amperes) since there is no direct contact between the nozzle and collector. Meanwhile, the voltages may vary from a few kilovolts up to 30 kV, which builds up a difference in the electrical potential between the nozzle and the collector, creating an electric field. Hence, safety during the working of the system should be of high priority. The working mechanism of electrospinning is based on this electric field. For the nanofibers to form uniformly in diameter, the applied voltage has to be maintained stably over time. The higher applied voltage can result in the material solution being pulled away from the nozzle faster. Thus, the applied potential is an important factor to be controlled, so that the solution droplet is stable in the cone-jet mode [28,29]. In this mode, the nanofibers can be generated with a more uniform diameter. By controlling the applied voltage, which can be easily optimized during the operation of electrospinning, the diameter of the nanofibers can also be controlled [30]. The higher the voltage, the smaller the diameter of the nanofibers. On the other hand, the diameter of the nanofibers is also highly affected by the material solution properties, system configuration, as well as environment, which is to be discussed in the following sections of the manuscript. However, there is no significant modification that can be carried out on the power source in the electrospinning system that can result in a change in the configuration of the nanofibers.

### 2.1. Polymer Solutions

#### 2.1.1. Solutions Using One Polymer

A solution with one or many polymers that are well dissolved in a low boiling point solvent can be used as material for the electrospinning process. The viscosity of the solution material can be considered the most important factor when operating an electrospinning system [31,32]. It can be affected by the following common factors: the molecular weight of the polymer, the functional structure of the polymer, and the viscosity of the solvent [33]. It is well known that the higher molecular weight of the polymer chain provides higher viscosity of the solution [34]. However, the contribution of the polymer molecular weight can be considered small, compared to the interaction between the polymer chains. The interchain interactions are induced by the functional groups of the polymer. Most of the interactions that are commonly encountered are hydrogen and van der Waals interactions. As the concentration of polymer increases, these interactions become stronger. Such a phenomenon can occur because shorter distances between the functional groups strengthen the interchain interactions. It was found that the solvent can also contribute to the viscosity of the solution, but this is also dependent on the interaction between solvent and polymer [35]. The viscosity of the polymer solution has a large impact on the shape of the electrospun fiber. The higher the viscosity, the harder it is for the solution to be split during the spinning process, and the larger the size of the outcome fibers. On the other hand, when the viscosity is too low, the solution quickly leaves the nozzle toward the collector as a drop of solution. It is noted that the electrical conductivity of the solution also affects the electrospinning process [36]. Table 1 suggests some works that use simple polymer solutions. While seeing that the simple polymer solution is the basic material for the electrospinning process, three groups of modification can be made: solutions using mixed polymer, block copolymer, and polymer composite, respectively, as described below:

#### 2.1.2. Solutions Using Mixed Polymer

The multiphase structures of the electrospun nanofibers are formed through the phase separations of the polymers in an emulsion solution. Even though the polymers are well mixed in the solvents, the surface tension between materials separates them from each other. Here, one of the polymers acts as the continuous phase (surrounding), while the other acts as the discontinuous phase (droplet). As the solvent is removed, the polymer hardens and forms many complicated structures [49,50]. At the nozzle, the discontinuous phase material is concentrated in the center, while surrounded by the continuous phase material. This process is named the self-assembly process, in which the materials reorganize themselves. In the case of electrospun nanofibers, polymers that exist within the solution will try to reorganize following the direction of the nanofibers. Note that surfactants can also be used to create an emulsion between hydrophobic and hydrophilic solutions (for example chloroform and water) for electrospinning [51]. Table 2 presents the emulsion mixtures of polymer solutions that can self-assemble to create multiphase electrospun nanofibers.

**Figure 2:**
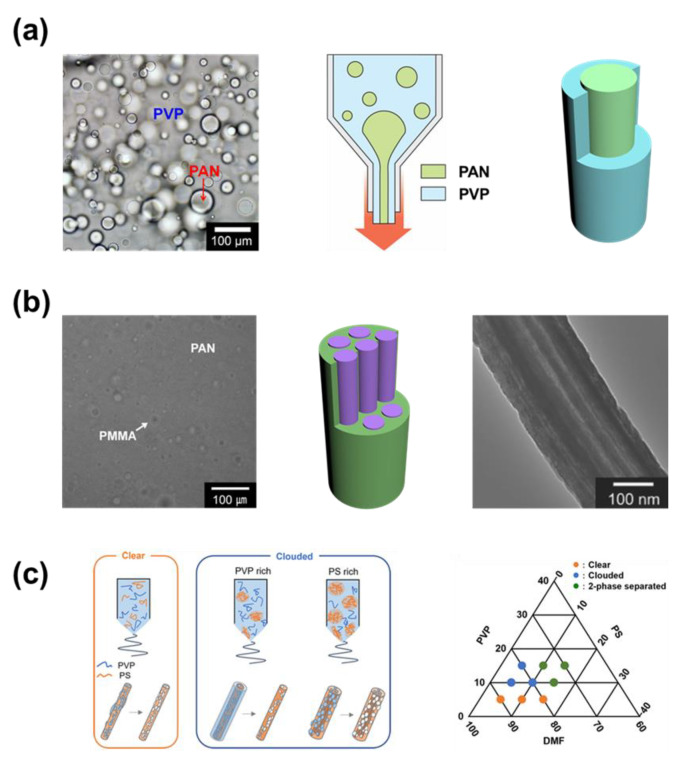
(**a**) Electrospinning process of PVP/PAN mixture, both are dissolved in DMF [52]. Copyright 2011, American Chemical Society. (**b**) The formation of PAN nanofibers containing PMMA multicore using electrospinning [53]. Copyright 2017, Wiley. (**c**) The effect of ration between PVP and PS over the formation of nanofibers with electrospinning [54]. Copyright 2022, American Chemical Society.

Lee et al. electrospun the solution containing a mixer of poly(acrylonitrile) (PAN) and poly(vinylpyrrolidone) (PVP) (Figure 2a) [52]. It has been proven that the viscosity of polymer components participating in the mixture can directly affect the disposition of polymers in the nanofibers. Here, the PVP was the continuous phase of low viscosity, while PAN was the discontinuous phase of much higher viscosity in the mixed solution. The constructed electrospun nanofibers contained a single large PAN core in the center covered by the PVP shell. However, when the mixed solution of poly(methyl methacrylate) (PMMA) and PAN was used for electrospinning (also demonstrated by Lee et al.), the structure of the nanofibers was much different (Figure 2b) [53]. The PAN was the continuous phase and covered the PMMA when the electrospun fiber was formed. Because the PMMA solution had lower viscosity compared to the PAN solution, the droplets of PMMA were very small in size and created multicore/multichannel inside the nanofibers. After the fiber was carbonized and PMMA decomposed, the PAN shell was carbonized into multichannel carbon nanofibers (MCNFs). Though the size of the channels was small, the structure with multichannel provided great porosity for the carbon fibers.

The ratio between the component polymers has been proven to be a decisive factor that affects their contribution in volume within the nanofibers. Narumi et al. electrospun the mixed solution of poly(styrene) (PS) and PVP in the N,N-dimethylformamide (DMF) solvent (Figure 2c) [54]. The authors investigated the structure of nanofibers by dissolving the PVP in the fibers with water. Note that there are polymer ratios that give phase separation, and these solutions cannot be used for electrospinning. When the PVP was the majority amount in the mixture, they covered the outside of the fibers, and PS formed a single core in the fibers. On the other hand, the PS-rich material formed fibers containing multi-PVP cores in the PS cover. In summary, the polymer of a larger amount usually covers the other polymer, though the structure of the core is highly dependent on the properties of the studied polymer.

**Table 2:** Combination of solutions, that can form the emulsion system for electrospinning.

Solution 1		Solution 2		Reference
Polymer	Solvent	Polymer	Solvent	
PEO	Chlorobenzene	PQT-12 ^1^	Chlorobenzene	[55]
PEO	DI water	Fibrinogen	Distilled water	[56]
Nomex ^2^	DMAc ^3^	Carboxylated nitrile butadiene rubber	Chloroform	[57]
PAN	DMF	PI ^4^	DMF	[58]
PPDO ^5^-b-PEG	Chloroform/DMF	PLA ^6^	Chloroform/DMF	[59]
Lysozyme/methyl cellulose	PBS ^7^	poly (DL-lactic acid)	Chloroform	[60]
PLGA ^8^/Span80 ^9^	Chloroform	FITC ^10^	DI water(or PBS)	[61]
PCL ^11^	Chloroform	Hyaluronan	Chloroform	[62]
PCL/Span80	Chloroform	PCL/Span80/MH ^12^	Distilled water	[63]
PHBV ^13^/Span80	Chloroform	PHBV/Span80/HF	Distilled water	

^1^ poly(3,3′-didodecyl quarter thiophene). ^2^ Poly(m-phenylene isophthalamide). ^3^ N,N-Dimethylacetamide. ^4^ Polyimide. ^5^ Poly(p-dioxanone. ^6^ Poly(lactic acid). ^7^ Phosphate buffer saline. ^8^ Poly(lactic-co-glycolic acid). ^9^ Surfactant. ^10^ Fluorescein isothiocyanate isomer. ^11^ Polycaprolactone. ^12^ Metformin hydrochloride (Drug). ^13^ Poly(3-hydroxybutyric acid-co-3-hydroxyvaleric acid).

#### 2.1.3. Solutions Using Block Copolymer

Another option for the material for single nozzle electrospinning is the use of block copolymers. Although there is no major phase separation, the self-assembly process can also occur at the nanoscale [64]. The volume fraction of the block copolymer will affect its structure after the self-assembly process [65,66]. Table 3 presents some of the block copolymers that have been used in electrospinning.

**Figure 3:**
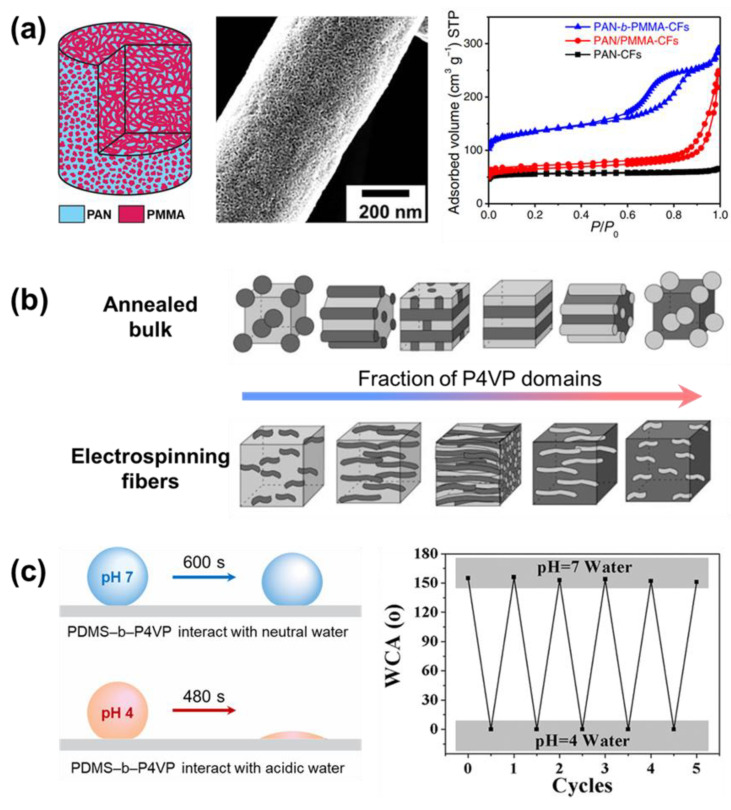
(**a**) The electrospun nanofibers fabricated from block copolymer of PAN and PMMA [67]. Copyright 2019, Science. (**b**) The effect of P4VP fraction in the block copolymer PSS-b-P4DP over the self-assembled structure of nanofibers [68]. Copyright 2007, Royal Society of Chemistry. (**c**) PDMS-b-P4VP, whose surface tension changed in contract with pH 4 and pH 7 water [69]. Copyright 2016, American Chemical Society.

Recently, Zhou et al. proposed carbon fibers that are the result of the carbonization of the PMMA−PAN block copolymer (Figure 3a) [67]. The fibers have been proven to contain microporous structures that can significantly improve the efficiency in the application of energy storage. However, a structure that has pores that are too small in size can bring certain disadvantages. For example, in the application of the capacitor (energy storage), the carbon material needs to be mixed with a polymer binder and conductive carbon black during device fabrication. In the study, the authors did not use any polymer binder or conductive carbon powder. The use of polymer binders can cover the porous structure, thereby greatly limiting the measured capacity.

Ruotsalainen et al. made a clear investigation of the block copolymer polystyrene-block-poly(4–vinyl pyridine) (PSS–b–P4DP) fraction’s ratios and their effect on the morphology of the fibers (Figure 3b) [68]. The proposed assembly structure can provide an excellent example of how, after the electrospinning process, the block copolymer assembles itself. As the ratio of the polymer fractions in the copolymer changed, the structure of the self-assembly material also changed. Thereby, the properties of the nanofibers may also be dramatically changed, both physically and chemically, together with their interface interaction. This phenomenon can greatly widen their field of application.

The different individual blocks in the block copolymer can exhibit their various properties independently. However, their interaction can contribute, and make the material suitable to be applied as a smart interface material. Li et al. introduced the electrospun block polymer poly(dimethylsiloxane)–block-poly(4–vinylpyridine) (PDMS–b–P4VP) as a switchable oil/water separate membrane (Figure 3c) [69]. Up point interacts with acidic water (potential of hydrogen, pH is smaller than 4), the poly(4–vinylpyridine) (P4VP) block of the polymer fibers stretches out and makes the surface of fiber highly hydrophilic. Meanwhile, the interaction with water with higher pH makes the poly(dimethylsiloxane) (PDMS) extend and stretch out. The PDMS at the surface makes the membrane fiber highly hydrophobic.

**Table 3:** The block copolymers that were used in electrospinning.

Block Copolymer	Solvent	Reference
PMMA−PS	DMF	[70]
PMTFPS ^1^−PMMA	THF/DMF	[71]
PEG−PCL	DCM ^2^	[72]
PEG−PLA	DMF	[73]
PS−PPG ^3^	DMF	[74]
PEO −PPO ^4^−PEO	Chloroform	[75]
PCL−PTHF−PCLPCL ^5^	Chloroform/methanol	[76]
PE−PVA ^6^/PLA	Chloroform	[77]
PS−PDMS ^7^/PS	THF/DMF	[78]
PLGA ^8/^antibiotic (fusidic acid and sodium fusidate)	THF/DMF	[79]

^1^ Poly[methyl(3,3,3-trifluoropropyl)siloxane]. ^2^ Dimethyl carbonate. ^3^ Polypropylene glycol. ^4^ Poly(p-phenylene oxide). ^5^ Polycaprolactone–polytetrahydrofuran–polycaprolactone. ^6^ Polyethylene-co-vinyl acetate. ^7^ Polydimethylsiloxane. ^8^ Poly(D,L-lactic acid-co-glycolic acid).

#### 2.1.4. Solutions Using Polymer Composite

**Figure 4:**
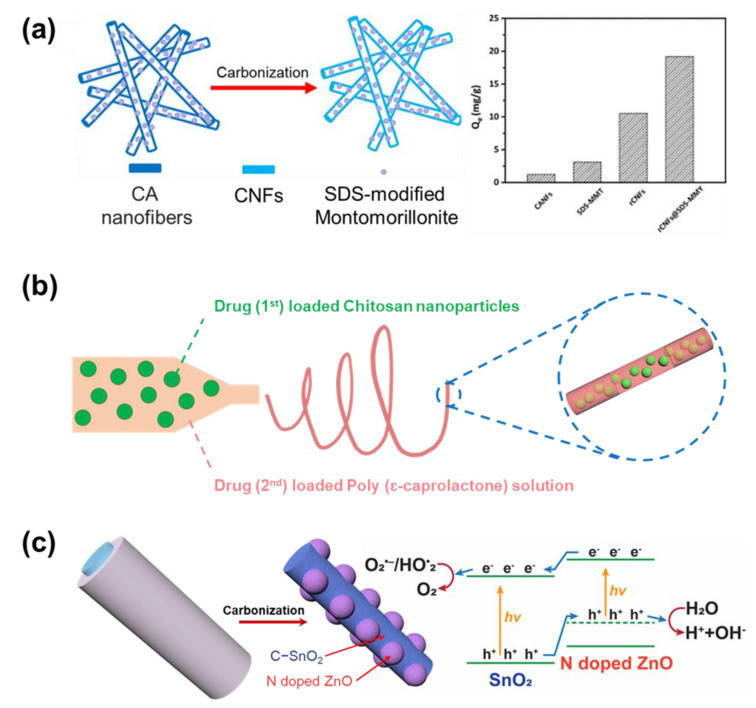
(**a**) The nanofibers composite of carbon and surfactant sodium dodecyl sulfonate filler [81]. Copyright 2017, Elsevier. (**b**) The fabrication of chitosan/poly (ε−caprolactone) nanofiber composite nanofibers for the application of drug delivery [82]. (**c**) N−doped ZnO nodule attached to the carbon nanofibers contains SnO_2_, the special structure that greatly enhanced the efficiency of catalyst activity [83]. Copyright 2012, Royal Society of Chemistry.

Another modification that has been carried out on the electrospun nanofiber, besides the selection of polymer materials, is the addition of fillers into the polymer matrix [80]. Though most of the common materials used in the electrospinning system are polymer solutions, other materials of limited amounts (organic, inorganic) can be mixed and spun together with the polymer. The results of such effort are the composite nanofibers, where the fillers are dispersed uniformly in the polymer matrix. The necessary condition for the formation of uniform fibers is that the filler must be mixed well in the solution, together with the polymer. Thus, the filler materials have to be chosen for their excellent interaction with the polymer and solvent (hydrophobic–hydrophobic or hydrophilic–hydrophilic). In the case where the fillers do not interact well with the solvent, the filler cannot disperse in the precursor solution. When the fillers do not have good interaction with the polymers, aggregation may occur while the solvent evaporates and the polymer reorganizes its structure. Table 4 presents the electrospinning processes that have been proposed for the fabrication of polymer composite nanofibers. Cai et al. introduced composite nanofibers that were constructed from cellulose acetate (CA) fibers and montmorillonite modified with surfactant sodium dodecyl sulfonate (SDS) (Figure 4a) [81]. The modification of the surfactant improved the dispersity of filler material in the polymer matrix, making a uniform structure for composite nanofibers. Thereafter, the fibers undergo carbonization, and CA turns to carbon nanofibers (CNFs). The results were products that have suitable properties to be used for heavy metal ions adsorption. The combination of CNFs and modified montmorillonite filler has been proven to significantly improve the performance of materials in the application.

Most of the applications where composite nanofibers are used have fillers playing the role of the main active material. Thus, the fillers should stay in the position favoring their activity. Wang et al. developed the chitosan/poly (ε-caprolactone) nanofiber composite used to store medicine and release it after a predicted period (Figure 4b) [82]. In the study, the chitosan particles that store the medicine chemicals were well covered in the polymer fiber, even if the particles were larger than the fiber diameter. This structure prevents medicine loss during the delivery process. On the other hand, there are applications where the fillers must have interacted with the electrolyte. For the material to effectively function, the particles should be concentrated on the surface of the fibers through a variety of fabrication methods. In the application of visible light catalysts, Lee et al. used a mixture of two solutions (PAN−SnCl_4_ and PVP−ZnAc_2_) to fabricate PAN composite nanofibers as the precursor for carbonization (Figure 4c) [83]. As demonstrated, the PAN formed a single core, while PVP formed the cover shell. After the carbonization, while PAN turned into CNFs containing SnO_2_, PVP decomposed completely and left the ZnO nodule attached to the wall of the fiber. The ZnO nodule greatly exposed to the electrolyte helps improve the electron exchanges, thereby creating the optimized condition for the activity of catalysts.

#### 2.1.5. Solutions Using Co-Solvent

In the electrospinning technique, a low boiling point solvent and a high boiling point solvent can be combined as co-solvents for the electrospinning solution. Because the vaporization rate of the solvent has a great impact on the formation of the polymer nanofibers, modification of the solvent can generate some special morphological structure for the material. On the other hand, the dependence of solvent vaporization rates on the environment can also vary, thereby opening the opportunity for the structure to be modified even further [95,96]. Though many results can be harvested from the modifications of co-solvent electrospinning, it also means that they are very sensitive to the fabrication conditions. Thus, they post many requirements for controlling the fabrication process. Table 5 presents some examples that used multi-solvent to modify the morphology of polymer nanofibers. In addition to that, phase separation that may occur between the solvents is a very important factor that needs attention during the operation of electrospinning. Phase separation can be triggered due to differences in the properties of solvents like hydrophilicity, surface tension, and electrical conductivity [97].

**Figure 5:**
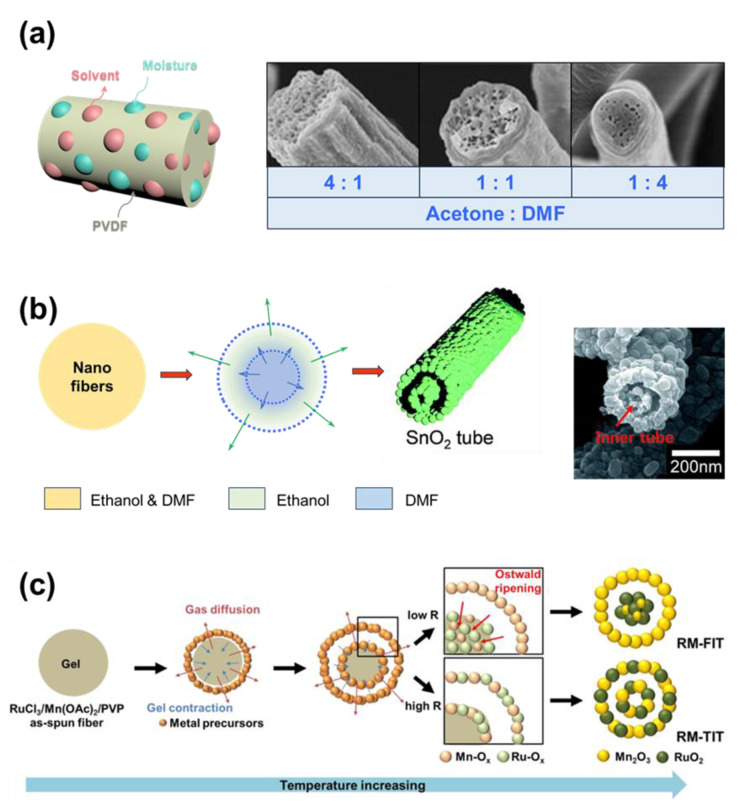
(**a**) Porous PVDF nanofibers, whose porous structure was created by the mixture of solvents (acetone and DMF) [98]. Copyright 2018, Springer. (**b**) The difference in evaporation rate of methanol and DMF solvent formed the tube-in-tube structure of SnO_2_ nanofibers [100]. Copyright 2017, Royal Society of Chemistry. (**c**) The effect of heating rate on the inner tube structure of the Mn_2_O_3_/RuO_2_ tube-in-tube [101]. Copyright 2016, American Chemical Society.

Zaarour et al. used the combination of acetone and DMF to prepare the solution for the production of electrospun polyvinylidene fluoride (PVDF) nanofibers (Figure 5a) [98]. While acetone is easy to vaporize, DMF is harder to vaporize. As the acetone vaporized, the water molecules were drawn into the fibers [99]. If the polymer fibers are spun in an environment with low humidity, the water droplets can quickly leave the fibers. Because PVDF cannot be dissolved in water, the trapped water molecule can create pores in the structure of nanofibers. However, the higher the humidity, the more difficult it is for the polymer matrix to release the water. Water nucleation growth occurs, and the water droplet can grow to a larger size. Finally, macro-size pores form in the structure and increase the size of fibers. In addition, the ratio of acetone over DMF affects the size of the pores and the polymer fibers’ diameter.

When there are fillers in the solution, special structures can also be formed. Lee et al. introduced a tube-in-tube structure of SnO_2_ that can be generated simply using a thermal treatment on composite nanofibers (Figure 5b) [100]. The PVP polymer and SnCl_2_ were dissolved with a mixture of DMF and methanol. After the solution was spun with the electrospinning technique, the nanofibers underwent the calcination process at 600 °C with a well-controlled heating rate. As the methanol quickly vaporized at lower temperatures, a layer of SnO_2_ formed at the outside wall of the fibers. Meanwhile, the DMF solvent vaporizes slower and forms another layer of SnO_2_ in the center. Note that PVP decomposed completely at high temperatures; thereby, no carbon is produced after the process. Here, the heating rate of the calcination process can decide the resultant shape of the SnO_2_ core layer. While the SnO_2_ molecules can rearrange themselves during a slow vaporization rate (slow heating rate) and the fiber solidifying, the high vaporization rate results in the hollow structure of the core. Meanwhile, Yoon et al. dissolved PVP in DMF and water together with RuCl_3_/Mn(OAc)_2_ (1:2) (Figure 5c) [101]. The RuCl_3_ and Mn(OAc)_2_ are the precursors of the RuO_2_/Mn_2_O_3_ complex. During the calcination process, the DMF quickly vaporizes, forming a hollow metal oxide outer shell, since it is the first solvent to vaporize. Water is vaporized at higher temperatures, and finally forms the core of the metal oxide fibers. In this study, the heating rate of the calcination process also plays an important role in the formation of the morphology of the fibers.

**Table 5:** The electrospinning solutions used the difference between solvents to alter the morphology of nanofibers.

Polymer	Solvent 1	Solvent 2	Reference
Poly (2-hydroxethyl methacrylate)	Formic acid	Ethanol	[102]
PS	DMF	Diethyl Formamide	
Nylon 6	HFIP	DMF	[103]
Nylon 6/NLS ^1^	HFIP	DMF	
PVC ^2^	THF ^3^	DMF	[104]
PLA	DCM	Ethanol	[105]

^1^ Montmorillonite. ^2^ Polyvinyl chloride. ^3^ Tetrahydrofuran.

### 2.2. Nozzle

#### 2.2.1. Single Steel Nozzle

As mentioned, the nozzle is one of the main components of the electrospinning system. That is the medium to transport the potential charge from the power source to the solution. The nozzle is usually made from stainless steel to prevent any unwanted reaction while having high electrical conductivity. The used nozzle can have different sizes depending on the viscosity of the material solution; the high-viscosity solution with high surface tension can make it hard for the solution to flow through a small sized nozzle. However, the nozzle with a higher gauge (smaller size) can produce smaller nanofibers. With one single nozzle acting as the only output for the material solution, there are fewer parameters to control. However, the spinning process can be conducted stably, and uniform nanofibers can be collected. Therefore, to date, it remains a favorable technique in the electrospinning process. Note that many simple nozzles can be used at the same time, and each of them can be provided with different polymer solutions [106,107]. The results are a mixture of different nanofibers with different properties, both physical and chemical, laid on top of one another. Though separated, the fabricated fibers can have amazing interactions. Ding et al. first used electrospinning to fabricate a layer of polyethyleneimine (PEI) fibers, which was used as a support for the much smaller nanofibers of polyamide 6 (PA) [108]. The diameter of the PA fibers is only around 30 nm. With the large size of PEI fibers, the distance between the PA fibers networks was greatly enhanced. The performance of PA nanofibers in the application of gas sensing is greatly improved since the gas can easily penetrate and interact with the sensing material. However, as the need for more complicated structures has increased, modifications have been made to create special structures for the fibers. With these modifications, many polymers can coexist within the nanofibers while being separated in phases, even if they are hydrophobic–hydrophilic polymers.

#### 2.2.2. Coaxial Nozzle

**Figure 6:**
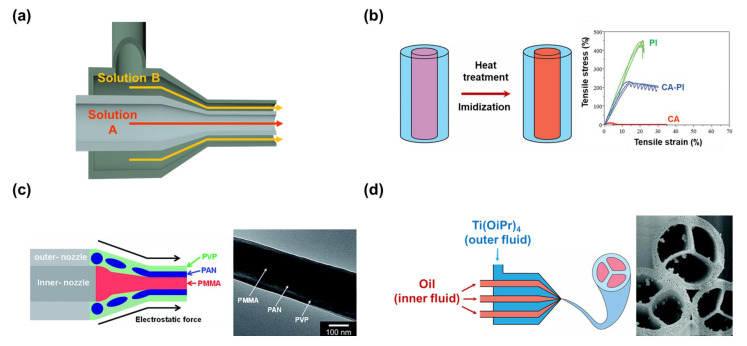
(**a**) The structure of a coaxial nozzle. (**b**) The fabrication of CA nanofibers with the core of polyimide [112]. Copyright 2016, Royal Society of Chemistry. (**c**) The nanofibers with three layers PMMA (core), PAN (middle), and PVP (shell) created with coaxial nozzle [113]. Copyright 2017, Royal Society of Chemistry. (**d**) Nanofibers whose controllable number of channels can be controlled with the number of inner nozzles [116]. Copyright 2007, American Chemical Society.

The coaxial nozzle (dual nozzle) is a special nozzle, where a larger nozzle covers a smaller nozzle (Figure 6a). The nozzle can give fibers a structure that has many layers, called core–shell nanofibers. The two nozzles in the coaxial nozzle are fed with two different polymer solutions, and the feed rates can be individually controlled. Yet, the feed rate is an important condition that has to be well controlled, so that the fibers can be generated uniformly. The method is normally used for two polymer solutions that are not intended to be mixed. The usual results of the technique are fibers with a single large core covered in a shell layer. The inner material can be removed to create a large channel in the center of the fibers or be the skeleton providing excellent physical properties for the fibers [109,110]. The number of layers can also be increased in number, creating wider choices for modifications [111]. Table 6 presents some combinations of solutions that were chosen for the coaxial nozzle.

In the application of oil–water separation, the membrane material must have good physical properties. The coaxial nozzle was used by Ma et al. to fabricate the fibers with the polyimide (PI) as the core, and CA as the shell of the fibers (Figure 6b) [112]. As it is nearly impossible to dissolve PI, poly amide acid (PAA) solution was synthesized, and used for electrospinning. After the membrane was collected, it went through the heating treatment, where the imidization can occur, and PAA turned into PI. The PI core plays the role of the skeleton providing excellent physical strength for the membrane. With the coating of fluorinated polybenzoxazine (F−PBZ) functional layer, the working efficiency in the separation of oil from water was improved, while maintaining the advantage of CA−PI fibers.

However, the size of the outer nozzle has to be large enough to cover the inner nozzle, leading to the large diameter of fibers. The large diameter of fibers may be a disadvantage of this nozzle set, reducing the surface area of the nanofibers. Lee et al. sought to reduce the size of the fibers by using a mixture of PAN and PVP as material for the outer nozzle, and PMMA as material for the inner nozzle (Figure 6c) [113]. The author used the coaxial nozzle to intentionally separate PMMA from the mixture of PAN and PVP. The product fibers have a structure containing three layers (PMMA/PAN/PVP). Since the PMMA was loaded into the inner nozzle, it became the core of the fibers. On the other hand, the mixture of PAN and PVP was divided into two layers, as previously mentioned. After the carbonization, the layers of PMMA and PVP decomposed completely, leaving the carbon material generated from PAN. The generated carbon fibers have both a channel and a small diameter compared to the diameter made from the other carbon fibers made from the coaxial nozzle [114,115].

The number of inner nozzles seems to be able to be increased to the desired number. For example, Zhao et al. surveyed how the number of inner nozzles affects the morphology of the nanofibers (Figure 6d) [116]. In the study, innocuous oil was used as the material for the inner nozzles, and PVP/Ti(OiPr)_4_ dissolved in ethanol was loaded into the outer nozzle. After the carbonization, TiO_2_ fibers with the desired inner channel were obtained as the oil, and the PVP decomposed. It is clearly shown that each inner nozzle loaded in the outer nozzle is responsible for the formation of a single channel. Using this method, the number of channels in the nanofibers can be controlled. However, the number of channels that can be loaded seems to be limited, due to the relationship in size between the inner and outer nozzles.

**Table 6:** The works that used coaxial nozzle to create core–shell nanofibers.

Inner Solution		Outer Solution		Reference
Polymer	Solvent	Polymer	Solvent	
PEO	Aqueous acetic acid (50%)	Chitosan	Aqueous acetic acid (50%)	[117]
PVA/Ti(OiPr)_4_	Acetic acid/DMF	PVA/C_10_H_25_NbO_5_	DMF	[118]
PS−PAN	DMF	PAN	DMF	[119]
PVA	DMF/ethanol	PVDF	DMSO/acetone	[120]
PS−PAN	DMF	PAN	DMF	[121]
PVP	Ethanol	NaCl	DI water	[122]
PEG	DI water	PLLA ^1^	DCM/DMF	[123]
C_16_H_30_O_4_Sn	Light mineral oil	PVP/Ti(OiPr)_4_	Ethanol	[124]
Tetrabutyltin	Mineral oil	PVP/Ti(OiPr)_4_	Acetic acid and ethanol	[125]
Nanosilver	Mineral oil	PVP/Ti(OiPr)_4_	Acetic acid and ethanol	[126]
	Mineral oil	PAN	DMF	[127,128]

^1^ Poly(l-lactic acid).

#### 2.2.3. Side-by-Side Nozzle

**Figure 7:**
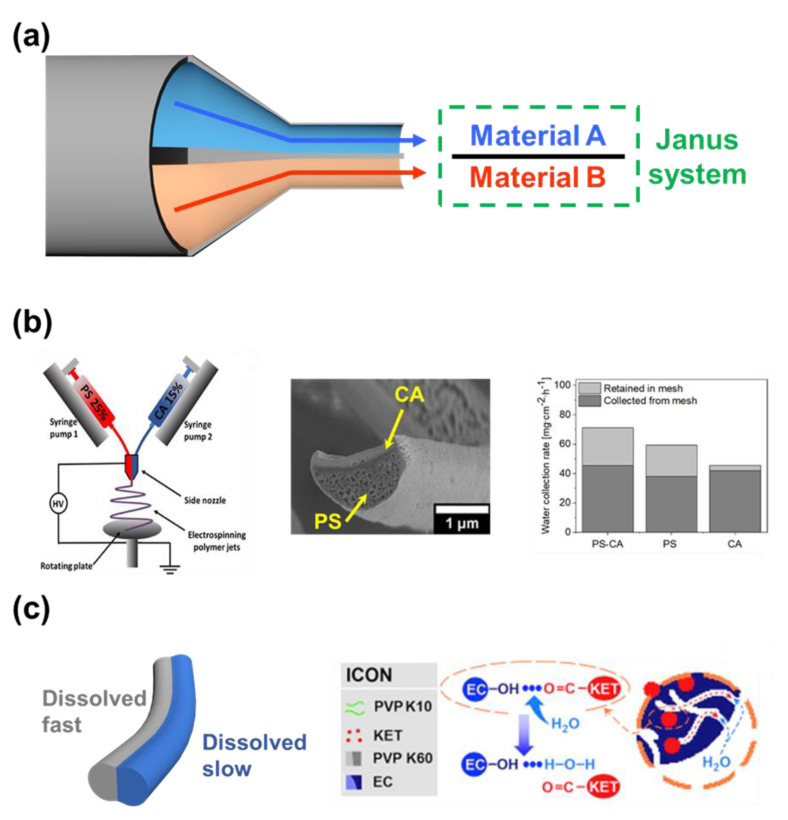
(**a**) Janus system created by electrospinning system using side−by−side nozzle. (**b**) Janus nanofibers consist of PS can CA, which was able to increase the efficiency in water harvesting [130]. Copyright 2021, American Chemical Society. (**c**) The different dissolve rates between the two sides of Janus nanofibers for drug delivery to multiple locations [132]. Copyright 2016, Elsevier.

The purpose of the side-by-side double nozzle is to fabricate a Janus interface material (Janus system), where the two materials with different wettability are attached to each other (Figure 7a). The electrospinning nanofiber now has both the advantage of a Janus system, and the introduction of excellent surface area of nanofibers structure to the potential application [129]. On the downside, the two polymer solutions leave the nozzle near each other, not as one. There is the chance that they will be drawn into the collector separately, and form two separate fibers. Both the feeding rate and the voltage applied to the nozzle need to be well controlled.

Knapczyk–Korczak et al. introduced Janus nanofibers constructed from PS and CA using side-by-side nozzle electrospinning (Figure 7b) [130]. In the water harvesting application, the nanofibers with extraordinarily high surface area greatly improved the water collection rate of the membrane material. The hydrophilic material can collect the water molecule effectively, yet the water droplet needs to overcome the resistance to fall off the surface [131]. The proposed Janus material constructed from a hydrophobic (PS) and a hydrophilic (CA) can collect the water droplet, and continuously drain away the water to the collector. On the other hand, the Janus nanofibers were used by Yu et al. as the base material for the delivery of drugs to multi-location (Figure 7c) [132]. By changing the composition of the load materials, the authors could control the time it took for the polymer matrix to dissolve in the aqueous environment. In these Janus fibers, the PVP side was dissolved quickly together with the drug when in contact with water. Ethyl cellulose (EC) was added to the PVP on the other side of the fibers to prolong the dissolving time, thereby allowing the drug to be released in the second location. However, because EC cannot be dissolved, a small percentage of the drug was trapped, and could not be released.

#### 2.2.4. Multiple Nozzle

**Figure 8:**
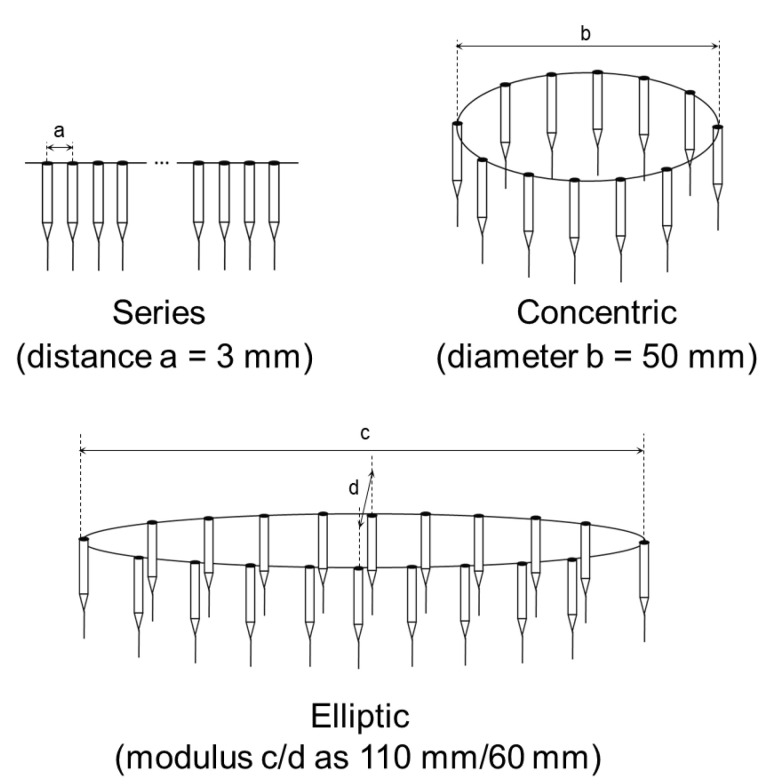
Some types of multi-jet electrospinning heads, with a is the distance between nozzle in series, b is the diameter of concentric, and c/d are modulus of ellipse alignment [136].

In industrial production, multiple nozzles and nozzles with less electrospinning can be used to increase the production rate, but excellent control is required for the uniformity of the nanofibers [133,134]. Multiple-nozzle electrospinning is an approach to enhancing the production rate of nanofibers. The working mechanism is based on traditional single-nozzle electrospinning but uses a combination of nozzles arranged according to specific geometries. The interaction between similarly charged jets from multi-nozzle electrospinning causes a repulsive phenomenon, leading to uneven fiber deposition and impacting the quality of the produced nanofibers. The repulsion phenomenon can be solved by increasing the distance between nozzles, so various configurations of multi-nozzles have been studied [135]. Tomaszewski et al. suggested three types of multi-jet electrospinning heads, including series, elliptic, and concentric. Among them, the concentric electrospinning head turned out best in both the efficiency and quality of the process (Figure 8) [136]. In addition, the drafting effect of the electric field force plays a key role in the formation of nanofibers. Then, Angammana et al. conducted a study about the correlation between the arrangement and the strength of the electric field [137]. They indicated that field distortion at each needle tip increases with the addition of needles. Furthermore, for the multi-nozzle electrospinning process to proceed smoothly, a higher voltage than usual must be used due to the large mass of the spinning solution delivered [138].

In addition to enhancing the fiber production rate, the multiple-nozzle system makes it possible to fabricate fibers from different materials simultaneously [139]. However, despite the efforts to improve multi-jet electrospinning that have been made, electrostatic field interactions between the needles and needle clogging are still the limits of this technique.

#### 2.2.5. Nozzle-Less

**Figure 9:**
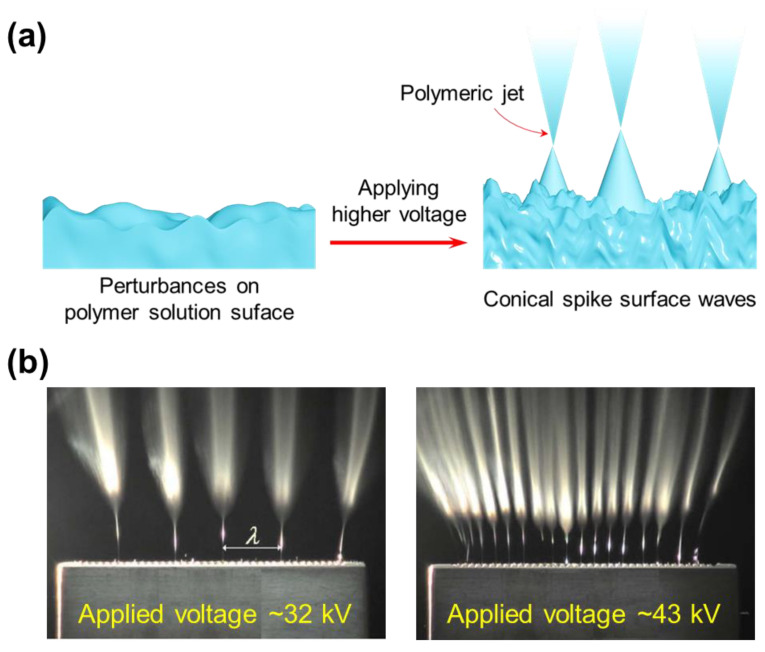
(**a**) Schematic illustration of multi-jet formation process on the free liquid surface. (**b**) The influence of the electric field on the number of polymeric jets [141]. Copyright 2008, AIP Publishing.

To address some drawbacks of nozzle-based electrospinning systems, nozzle-less electrospinning has been developed. Theoretically, the polymer jets of this system are generated by an external force (high voltage) added to create perturbances and obtain conical spike surface waves like Taylor cones [140]. A nanofiber web can be created once the polymeric jets have reached a conductive collector (Figure 9a). It should be noted that the concentration of electric force on the solution surface is a crucial issue for needleless electrospinning. The influence of the electric field on polymeric jets was demonstrated by Lukas et al. (Figure 9b) [141]. With the increase in voltage, the number of jets produced will increase. In addition, the nozzle-less electrospinning systems are sensitive to various parameters, such as the surface tension of the polymeric solution, the distance between the collector and the solution surface, etc. There are numerous needleless electrospinning architectures that have been designed, which focus on solution reservoirs, rotating spinnerets, syringeless, and so on [142,143].

By loading extremely high voltages on the free liquid surface, numerous jets can be obtained simultaneously, which means a high production capacity can be achieved in the nozzle-less electrospinning technique. Moreover, obstacles caused by nozzles are absent in this system, like electrospinning of colloidal suspensions or needle clogging [144]. However, the relatively complex setup, applied high voltage, and hard-to-produce advanced nanofiber structures are still disadvantages. In addition, further studies need to be conducted on the fiber quality control and electrospinning processes before they reach industrialization and commercialization.

### 2.3. Collector

#### 2.3.1. Planar Collector

Planar is the simplest structure of a collector, where a metal plate is used as a collector. Though the structure is simple, most of the fibers can be collected without the loss of material. The collected fibers are arranged in random directions. However, there are situations where the fibers are swollen. In this case, a large amount of solvent is hard to vaporize during the spinning process, which can be caused by the environmental condition, or the component of the electrospun solution. The remaining solvent pulls the surrounding polymer materials together and slowly dissolves them. To overcome this limitation, another type of collector has been developed.

#### 2.3.2. Rotational Collector

**Figure 10:**
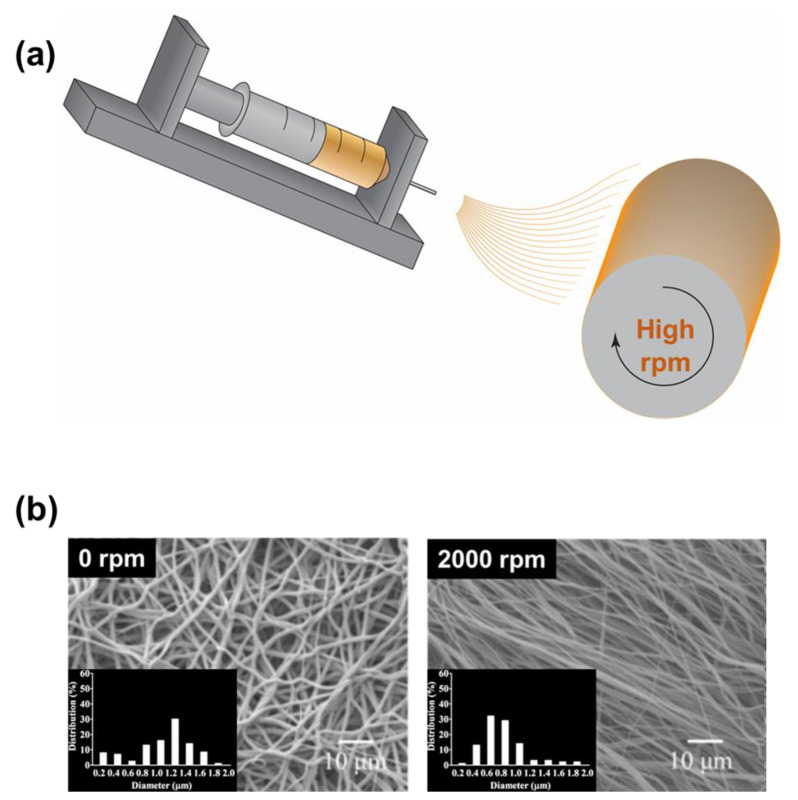
(**a**) Electrospinning system with the rotational collector. (**b**) The well-aligned nanofibers collected by using a drum-type collector [145]. Copyright 2017, Elsevier.

The rotational collector (drum-type collector), whose working mechanism is similar to that of a winder machine, can be used to collect the electrospun nanofibers (Figure 10a). The drum operates with a high-speed rotation to stretch the electrospun fibers, preventing their swelling. During the rotation, the air also circulates much better, thus the solvent evaporation rate was significantly improved. Thus, the drum collector can be used when it is difficult for the nanofibers to be collected by a planar collector. On the other hand, the nanofibers collected by the drum can be well aligned in one direction, whether it is intentional or not. Manuel et al. produced nanofibers for drug delivery with a drum-type collector (Figure 10b) [145]. The field emission scanning electron microscope (FE-SEM) figures indicate that the material was clearly highly aligned. However, the limitations of the drum-type collector come with the high-speed mechanical movement. Regular maintenance needs to be carried out more often to ensure the safety and stability of the rotation. Energy consumption for the drum is another issue that needs to be addressed, considering that the electrospinning operation already requires high power.

#### 2.3.3. Three-Dimensional Structure Collector

The nanofibers that were collected by the traditional planar collector can have a very random orientation. However, some of the advanced applications can have specific requirements for the structure of the membrane material. Many efforts have been made to align the electrospun nanofibers following a designed pattern.

**Figure 11:**
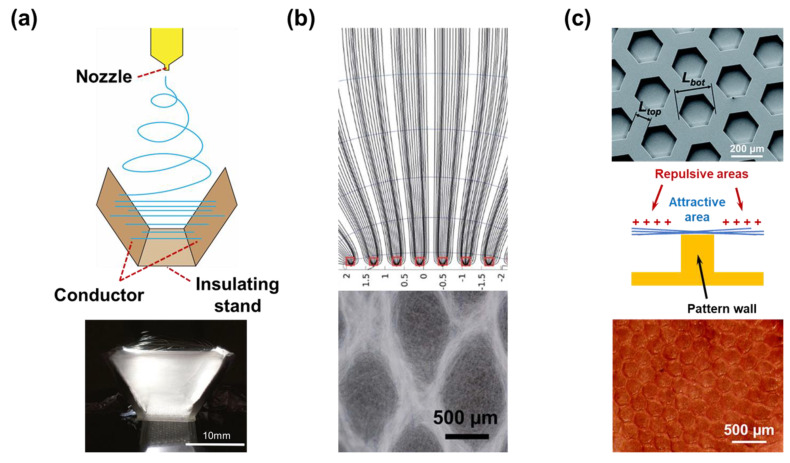
(**a**) Three-dimensional collector for the collection of the one-direction aligned nanofibers [146]. Copyright 2018, Springer. (**b**) Nanofibers align following the pattern created by conducting and non-conducting layers of collector [148]. Copyright 2013, Royal Society of Chemistry. (**c**) Three-dimensional conducting collector with a pattern wall, which created an attractive force for the alignment of nanofibers [149]. Copyright 2015, Royal Society of Chemistry.

Because of the limitation in aligning fibers using a drum-type collector, Tan et al. proposed a method to align the electrospun nanofibers using a 3D collector (Figure 11a) [146]. The study provided highly detailed information about the method to generate mono-directional aligned nanofibers for tissue engineering. Instead of using a single planar collector lining on the ground, two conducting collectors were placed in a V shape. Knowing that the collector position can be an important factor in the study, the collectors were assembled at two angles of 45° and 60°. The results from the 45° collector showed better control over the orientation of the nanofibers. The orientation of the nanofibers only concentrated around the angle of 90° at the specific height of 7.7 mm from the bottom of the collector. However, because the electrospun material did not stack on top of each other, the interaction between the nanofibers was reduced. The potential to be gathered as a membrane for use in other applications may be put into question.

It was declared that topography is still one of the most critical conditions for cell culture, especially stem and bone tissues [147]. To date, chips have been fabricated using very effective photolithography, etching, or molding techniques. However, to mimic the in vivo extracellular matrix, which is constructed from nanofibers composite, Zhao et al. electrospun and aligned the nanofibers following the designed pattern with the 3D pattern collector (Figure 11b) [148]. The authors investigated the interaction between the conducting and non-conducting layers in the patterned collector. The non-conducting layer created a repulsive force, while the conductive layer created an attractive force towards the nanofibers. By patterning the layers, repulsive and attractive areas can be designed, and the resultant is the pattern of the nanofibers membrane. Furthermore, a single 3D conducting patterned collector was used by Nedjari et al. to make the electrospun nanofibers align following a pattern (Figure 11c) [149]. Though there is no non-conducting area on the pattern, attractive and repulsive areas can still be formed. The electrospun nanofibers leave the nozzle carrying electric charges, but the charges are released when making contact with the attractive pattern wall of the collector. The nanofibers that cannot make direct contact with the collector still have the changes, thereby creating repulsive forces in the valley between the walls. As a result, the latter electrospun nanofibers are only concentrated on the top of pattern walls. The developed method is easy to execute, but as the membrane becomes thicker, the repulsive area is less effective. Thus, the thickness of the fabricated membrane seems to be limited.

#### 2.3.4. Bath Type Collector

Though most of the nanofibers should be collected in a dry environment, there are some exceptions. In some cases, the electrospun nanofibers can be collected in a bath collector containing liquid base material.

**Figure 12:**
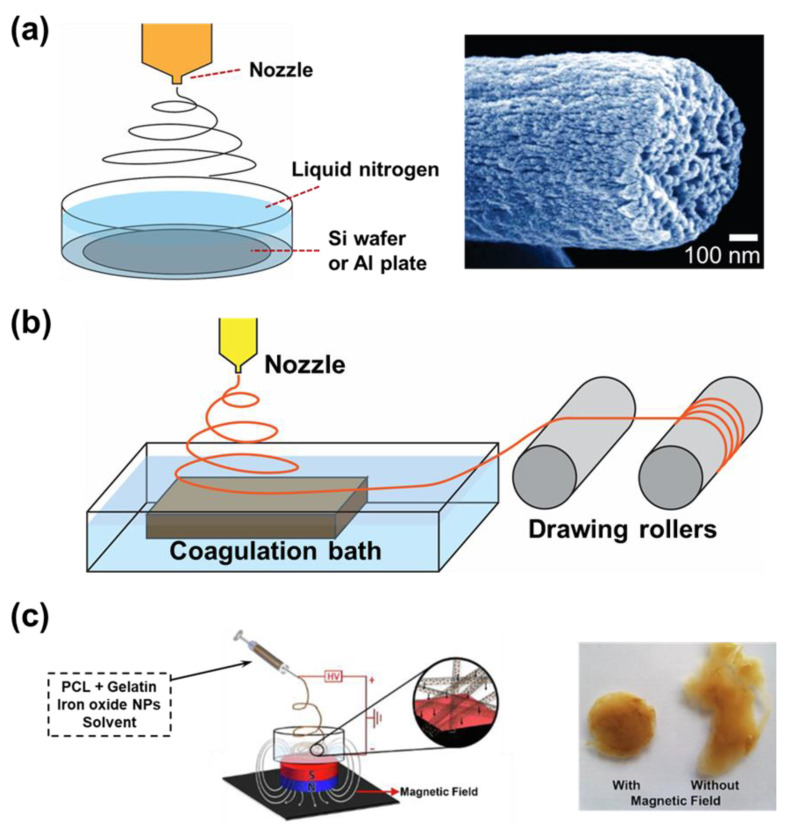
(**a**) The porous PAN nanofibers created by quickly freezing the newly electrospun fibers with liquid nitrogen [150]. Copyright 2006, American Chemical Society. (**b**) The electrospinning of poly amid 6/66 into a water bath, which was later collected with drawing rollers [151]. Copyright 2018, Springer. (**c**) The nanofibers contain iron oxide nanoparticles for easy collection from water baths using a magnetic bar [152]. Copyright 2022, Elsevier.

Many methods have been proposed to generate porous structures for the polymer fibers. However, these methods usually require sacrificial materials, but the removal of the sacrificial materials can also affect the main materials. McCann et al. electrospun a PAN solution directly into a bath of cryogenic liquid (nitrogen liquid) to create highly porous PAN nanofibers (Figure 12a) [150]. Before the solvent evaporated, the temperature of the polymer suddenly dropped lower than its glass transition temperature. The cool environment separates the polymer-rich and solvent-rich phases from each other, which is the driving force forming the polymer porous nanofiber. Thereafter, the polymer nanofibers need to warm up rapidly in the air to stabilize the fibers.

A variation of electrospinning is sol–gel electrospinning, where the nanofibers still hold solvents inside the fibers. One example of sol–gel electrospinning was when the poly amid 6/66 (dissolved in formic acid and acetic acid) was used as the material. To overcome the limitation, Franco et al. spun the nanofiber directly into a bath containing distilled water (Figure 12b) [151]. The solvent was freed from the polymer matrix by the dispersion of solvent into the water, which can occur at a much higher rate than evaporation. Because the solvent was suddenly removed from the nanofibers, the solid polymer nanofibers have a porous structure, even though the porosity seems to be small. Finally, the electrospun fibers have to be collected by a drawing roller system. The difficulty in collecting the fibers from the water bath brought many limitations in application. However, it is undeniable that the wet electrospinning technique can create distance between the layers of nanofibers. This structure can offer many advantages. For example, in the studies of tissue engineering, it allows the cells to penetrate and migrate. Bakhtiary et al. introduced a method to use a magnetic field to collect the nanofibers in a water bath (Figure 12c) [152]. The author mixed superparamagnetic iron oxide nanoparticles into the polymer solution. Under a magnetic field, the nanofibers containing particle material were drawn towards the magnetic bar placed under the bath. After freeze-drying, aerogel-like material could be collected, and later used for cell seeding.

#### 2.3.5. Electrode-Assisted Collector

**Figure 13:**
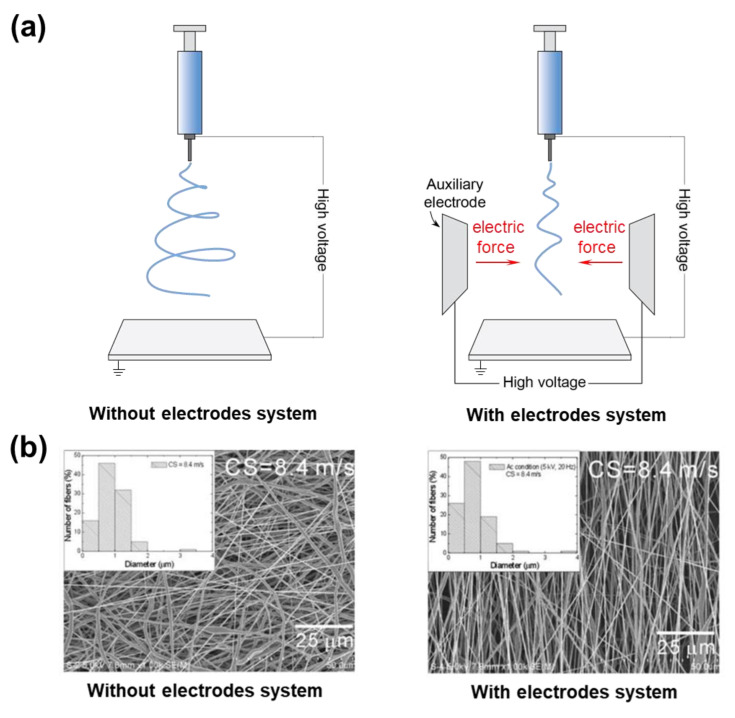
(**a**) Schematic illustrates the bending instability of a polymer jet with and without auxiliary electrodes. (**b**) The fiber deposition onto a rotating collector with and without auxiliary electrodes [155]. Copyright 2009, Springer.

To intensify the electric field and enhance jet stabilization in the electrospinning system, auxiliary electrodes are employed, which control the deposition and diameter of electrospun nanofibers [153]. Ring, cylindrical, and plate are the most popular shapes of auxiliary electrodes. Normally, the auxiliary electrodes are added between the needle and collector and carry a similar polarity to the needles (Figure 13a). By increasing the applied potential of electrodes, the electrospinning jet is narrowed, limiting the area of fiber deposition. In addition, many researchers have studied the aspect of materials, type of polarity, type of supplied power (AC or DC), and location of the electrodes in the set-up to obtain the fiber morphology that meets the needs of the final application [154]. Moreover, it is possible to control fiber orientation and obtain aligned fibers by modification of the collector of this electrospinning system. For instance, by using a rotating collector and adjusting its surface speed, highly aligned polymer fibers can be obtained [155] (Figure 13b).

#### 2.3.6. Magnetic Field-Assisted Collector

**Figure 14:**
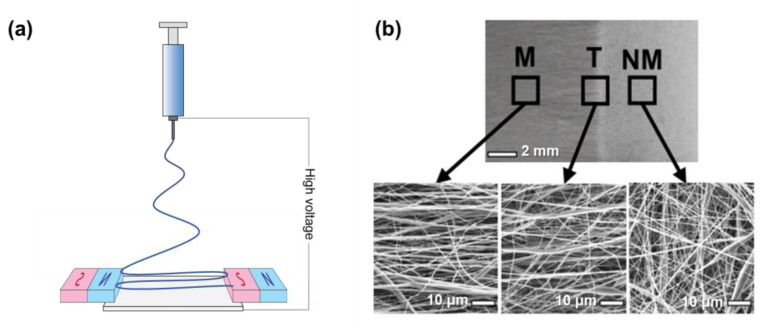
(**a**) Schematic illustrates the magnetic field-assisted electrospinning system for preparing aligned nanofibers. (**b**) Electrospun fiber alignment in magnetic field (M), transition (T), and non-magnetic (NM) region [158]. Copyright 2023, Wiley.

Other than the electric field derived from auxiliary electrodes, a magnetic field derived from permanent magnets can also be used to assist the electrospinning to produce highly aligned fibers. The most common setup is a pair of permanent magnets installed between the collector of the conventional electrospinning with no direct contact between them (Figure 14a). When the whipping jet approaches the collector, under the influence of the magnetic field, it is stretched and aligned across the magnets [156]. To enhance the spatial control ability and fiber morphology, several parameters of magnetic field-assisted electrospinning have been investigated, including magnet geometry, voltage, magnetic nanoparticle presence in the polymer solution, and flow rate [157]. Recently, magnetically assisted electrospinning allowed precise spatial control over electrospun fiber alignment for fabricating musculoskeletal interfacial tissues that have complex gradient structures. Here, the collector was designed with three regions that have different magnetic properties, including magnetic (M), non-magnetic (NM), and transition (T) (Figure 14b) [158]. As a result, electrospun fibers were highly aligned in a magnetic field and randomly aligned within the non-magnetic region. It is also indicated that magnetic field-assisted electrospinning is possible for spatial control over fiber alignment, even at sub-millimeter resolution, showing a bright future for this method in tissue engineering.

### 2.4. Other Technical Factors

#### 2.4.1. Distance from the Nozzle and the Collector

**Figure 15:**
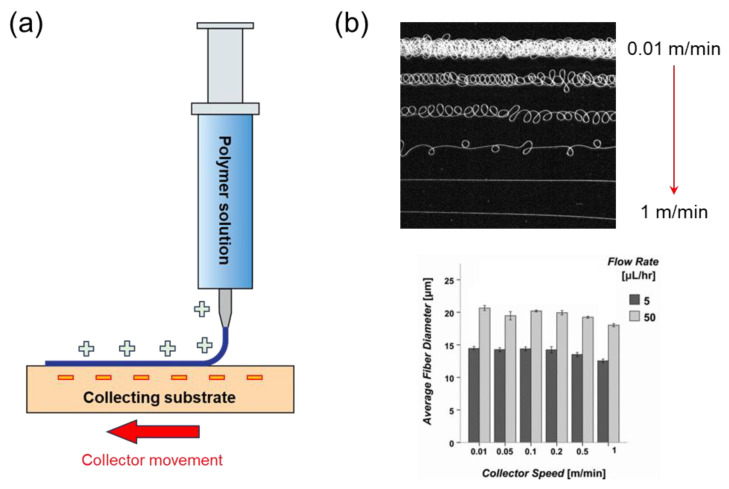
(**a**) Near-field electrospinning system and (**b**) the effects of flow rate and collector movement speed on the deposition of electrospun nanofibers [160]. Copyright 2011, Wiley.

Nearfield electrospinning was designed to completely overcome the shortcomings in random nanofibers generated by traditional electrospinning systems. Here, the distance between the nozzle and the collector is dramatically reduced (Figure 15a). It was suggested to be maintained from 0.5 to 3 mm [159]. Instead, the nozzle or collector can be mobilized enabling the ability to be deposited over long distances, which is similar to the 3D printing technique. This speed plays an important factor in the operation of the near-field electrospinning in addition to the mentioned parameter. As the movement speed becomes faster, the generated nanofibers will be stretched into a single straight fiber (Figure 15b) [160]. These fibers can be deposited following a designed location. Despite the limitation in the slow production rate of near-field electrospinning, excellent accuracy in orientation provides potential in the application of nanodevice materials. For instance, well-oriented nanofibers can greatly contribute to controlling pore size, which is highly required in cell cultivation [161].

#### 2.4.2. Applied Voltage Types

**Figure 16:**
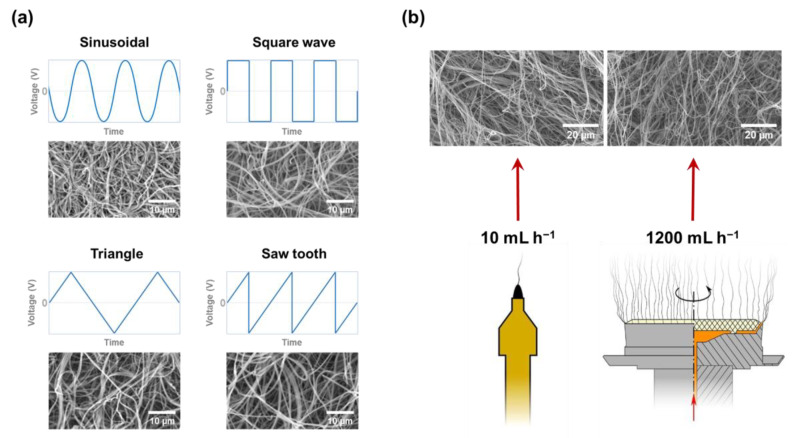
(**a**) The nanofibers generated from electrospinning using different waveforms of AC [162]. Copyright 2020, Elsevier, and (**b**) the stability in nanofibers formation in high feeding rate of AC electrospinning using large size rotating nozzle [163]. Copyright 2019, Elsevier.

Traditionally, the electrospinning technique has utilized direct current (DC). However, during the development of the electrospinning technique, alternating current (AC) was applied to limit the accumulation of electrical charge on the collected nanofibers. Here, the frequencies and waveforms of AC used for the electrospinning process were proposed in Figure 16a [162]. No clear differences can be detected from SEM images, whether in fiber diameter or uniformity. However, the efforts to vary the polymer concentration and feeding rate cannot fully overcome the limitation of AC electrospinning, which produces nanofibers with poor quality, large beads, and droplets. Here, the electrical conductivity of the solution can be considered a decision role to overcome this limitation. For instance, Farkas et al. investigate the contribution of polyvinylpyrrolidone K90 (PVPK90) and electrical conductivity, which is controlled by the amount of sodium dodecyl sulfate, to the stability of electrospun nanofiber formation (Figure 16b) [163]. Not only that, once the nanofibers can be generated uniformly from the electrospinning, the feeding rate can be increased up to 1200 mL h^−1^. Such high feeding rates open up the potential for electrospinning techniques on an industrial scale.

## 3. Applications

Electrospinning can produce outstanding candidate materials for a wide range of applications. However, there are many applications where the enhanced performance can be carried out by the modification in nanofibers morphology are few in number. Some of the representative applications can be named including air purification, sound absorption, heat retention, etc. [164,165,166,167,168,169,170]. Their development rather lies in the interactions between the fibers with each other to build the membrane network. On the other hand, electrospun nanofibers whose morphology has been modified have been proven to be able to provide suitable active sites for the operation of the following applications: oil/water separation, drug delivery, tissue engineering, etc. [171,172,173].

The after-treatment of the electrospun nanofibers also opens up the potential for more advanced applications. The most popular treatment is the carbonization process at high temperatures. There, electrospun nanofibers are converted into materials with different compositions depending on the carbonization conditions. The conditions that contribute to the properties and structure of the outcome material are the electrospun solution component, carbonized temperature, environment, and heating rate. While the polymer can act as the precursor that is converted into carbon material, it can also serve as the template for the structure formation of metal or metal oxide. Though the high surface area of the product nanofibers can improve the performance in the application by prioritizing the interaction between the active material and the working environment, their low physical properties limit their use in the mentioned applications. Instead, excellent electrical conductivities make them ideal materials for the development of applications involving electrochemical activities [24].

### 3.1. Oil/Water Separation

**Figure 17:**
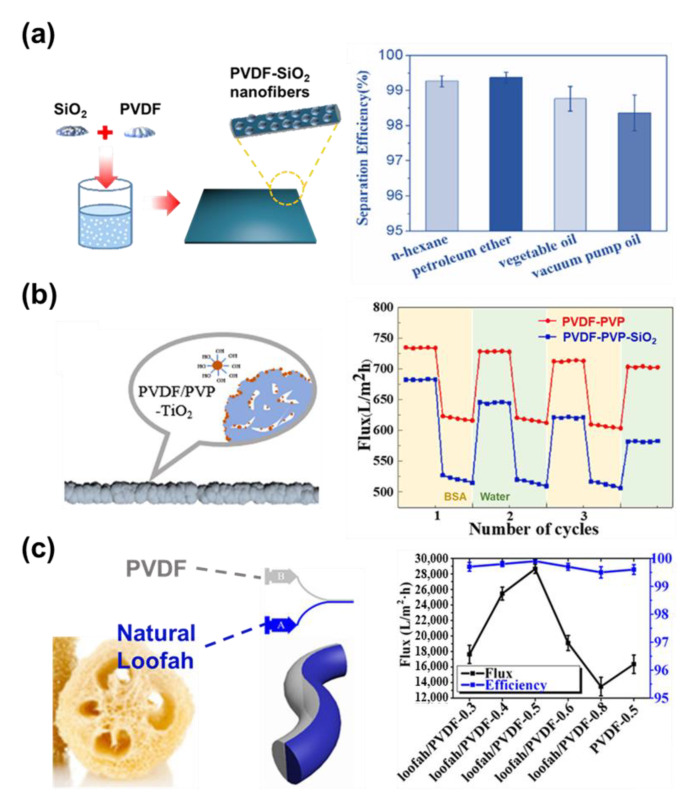
(**a**) The fabrication of PVDF-SiO_2_ nanofiber membrane and its application in oil–water separation [175]. Copyright 2020, Wiley. (**b**) Fabrication procedure of PVDF/PVP-TiO_2_ hydrophilic filter through one-step electrospinning method [176]. Copyright 2021, Elsevier. Copyright 2022, Elsevier. (**c**) Core–shell structure of loofah–PVDF membrane and its oil–water separation behavior [177]. Copyright 2020, American Chemical Society.

Oil/water separation is important for the protection of the environment from oil pollution and its deleterious effects [174]. Due to its facile process and various structural designs, the electrospun fiber membrane is considered a good material for the application of oil/water separation. These modifications mainly focus on improving the interaction between the surface of nanofibers and the surrounding environment. Loading inorganic nanoparticles into the hydrophilic polymer can greatly improve the hydrophilicity of the filter membrane. Jiang et al. manufactured an effective oil/water separation membrane through a one-step electrospinning process using a poly(vinylidene fluoride)-silica (PVDF−SiO_2_) blend solution (Figure 17a) [175]. The SiO_2_ nanoparticles also provide a rough surface to PVDF nanofibers, which increases the solid–liquid contact area, leading to higher geometric hydrophobicity. Because of the hydrophobic SiO_2_, the PVDF−SiO_2_ nanofibers membrane has super-hydrophobic and super-lipophilic properties, when compared to the pure poly(vinylidene fluoride) (PVDF) fibers. Consequently, the PVDF−SiO_2_ nanofibers exhibited a separation flux of 2963 L·m^−2^h^−1^ and a separation efficiency value of 99.4%, which indicates its suitability for use for oil/water separation. Du et al. represented a functional separation membrane through a one-step electrospinning method. They produced a PVDF/PVP−TiO_2_ hydrophilic nanofiber membrane by using a blend solution (Figure 17b) [176]. While PVDF as a base material can provide good mechanical strength and chemical resistance, PVP assists the hydrophilicity of the TiO_2_. The TiO_2_ allows membranes to have self-cleaning ability, pollution prevention, and antibacterial properties. Membranes of the three ingredients showed high separation efficiency for n-hexadecane, petroleum ether, and edible oil. Likewise, the PVDF/PVP−TiO_2_ nanofiber fulfilled high oil–water separation and anti-fouling properties.

In another example, Wang et al. produced a switchable oil–water separation membrane based on natural loofah and PVDF through side-by-side nozzles (Figure 17c) [177]. The difference in surface tension between the PVDF and loofah solution provides the driving force to form a core–shell structure. The natural loofah/PVDF nanofibers behave especially as a switchable oil/water separator that allows oil separation in dry conditions, and water separation in wet conditions. As such, the PVDF/loofah-based Janus membrane produced by side-by-side nozzles effectively achieves a core–shell structure for effective oil/water separation.

### 3.2. Tissue Engineering

Tissue engineering, known as regenerative medicine, has in recent years been developed rapidly as an effective treatment method for tissue injuries by providing the appropriate physiological microenvironment. One of the most important components of tissue engineering is the scaffold that supports the growth of cells and provides a vector that delivers the biochemical factors [178]. Because the different properties can be found in different kinds of tissue, the requirements of the scaffold can also be highly specific. In addition to the high porosity and the ability to be aligned, the wide choices of materials allow the electrospinning of nanofiber products with a diversity of properties. Therefore, electrospinning can be considered a powerful tool to manufacture scaffolds for tissue engineering.

**Figure 18:**
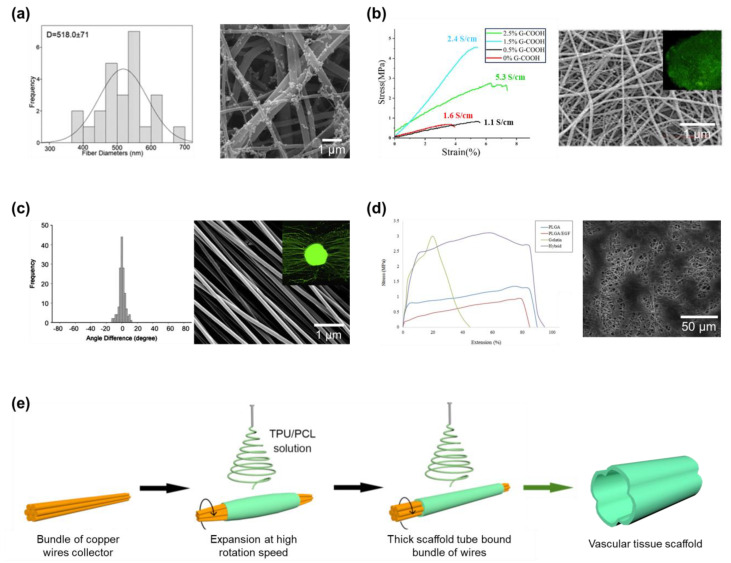
(**a**) High physical properties nanofibers for the scaffold of bone tissue engineering [179]. Copyright 2019, Elsevier. (**b**) Electrical conducting nanofibers as the scaffold of cardiac tissue engineering [180]. Copyright 2020, American Chemical Society. (**c**) Well-aligned nanofibers as scaffold for nerve tissue engineering [181]. Copyright 2010, Elsevier. (**d**) Highly stretchable electrospun membrane as scaffold of the skin tissue engineering [182]. Copyright 2014, Wiley. (**e**) Bundled copper wires collector for the fabrication of vascular tissue scaffold [183]. Copyright 2018, Elsevier.

In bone tissue engineering, the prioritized properties of the scaffold are the high porosity and mechanical properties of the membrane. Since electrospun nanofibers can construct a membrane with extraordinarily high porosity, fillers can be added to the nanofibers to improve their mechanical properties. In addition to the polyhydroxybutyrate (PHB) matrix in the nanofibers, Toloue et al. added chitosan and alumina nanowires as the filler for the nanofibers (Figure 18a) [179]. The composite nanofibers have their physical strength significantly improved, which makes them suitable to act as scaffolds for bone tissue engineering. The fillers also help stabilize the nanofibers in the aqueous environment, preventing the structure of nanofibers from collapsing during the construction period of tissue. For use in cardiac tissue engineering, the scaffold built from electrospun nanofibers must have good electrical conductivity and elasticity to mimic the cardiac function. Talebi et al. proposed the composite nanofibers built of a network of polycaprolactone, chitosan, and polypyrrole (Figure 18b) [180]. The membrane fibers have good electrical conductivity thanks to the graphene and polypyrrole segments. As more graphene was introduced into the material, the electrical conductivity was also enhanced. The scaffold material with more graphene contained also expresses outstanding mechanical strength, suitable to be applied in the application of cardiac tissue development. However, the amount of graphene filler being higher than 1.5% can significantly reduce the mechanical properties. In the scaffold for nerve tissue engineering, physical strength is no longer a priority. Here, the alignment of nanofibers needs to be the focus of attention. The alignment of the nanofibers in the membrane can guide the neurite extension through a lesion site for regeneration. Biocompatible PLA nanofibers were aligned following a single direction by Wang et al. (Figure 18c) [181]. The authors showed the positive effect of nanofiber alignment over the culture of nerve tissue and also presented the influence of the diameter of the fibers. On the other hand, the development of scaffolds for skin engineering should be considered angiogenesis, gas exchange, moisture maintenance, and the mass transport of tissue. Therefore, while electrospinning can provide a membrane constructed from nanofibers that have suitable properties, the scaffold still needs to have physical properties that match that of the native skin tissue, especially the high stretchability. Norouzi et al. used the hybrid between poly(lactic-co-glycolic acid) and gelatin as material for electrospinning (Figure 18d) [182]. The fibers could later be collected as a stretchable membrane (up to 90%) that can be used in skin tissue engineering and wound dressing. Until now, many efforts have been carried out to mimic vascular tissue. However, its own structure resembling a small diameter hollow tube is the biggest challenge that needs to be overcome. However, electrospinning can overcome such small size fabrication by reducing the diameter of the rotated drum collector. The well-oriented nanofibers can provide a suitable scaffold for the cultivation of vascular cells. However, one issue that remains during the production of electrospun scaffolds is that the removal of nanofibers can damage the microstructure. To overcome the limitation, Mi et al. modified the collector into a bundle of copper wires as demonstrated in Figure 18e for the fabrication of a tube scaffold [183]. When the rotating speed increased during the operation of the electrospinning system, the centrifugal force made the satellite copper wires expand affecting the initial diameter of the tube. Thereafter, the thickness of the tube bound the wire collector and created a wavy configuration on the inner layers. The reported nanofiber tube not only be easily collected, but its structure is also similar to the zero stress state of the blood vessel.

### 3.3. Supercapacitor

**Figure 19:**
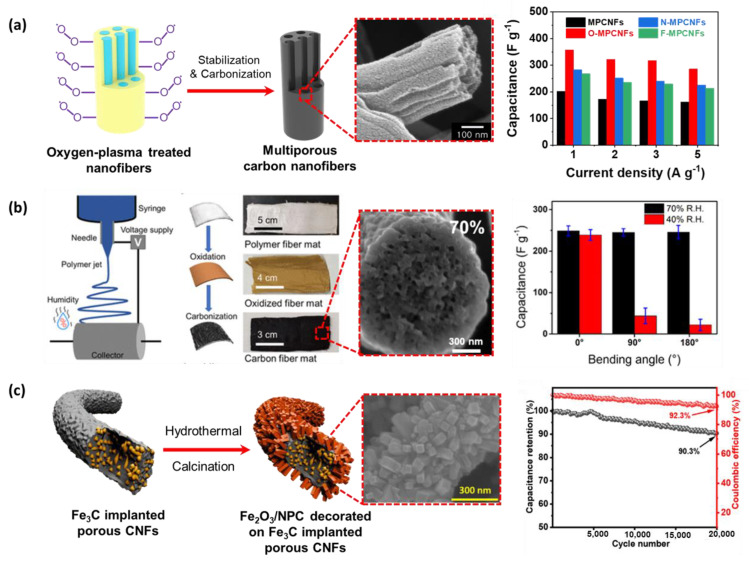
(**a**) The oxygen-plasma-treated multiporous CNFs showed the highest capacity value compared with other ones [184]. Copyright 2023, MDPI. (**b**) The PMMA−PAN fibers under a relative humidity of 70% and its gravimetric capacitance [185]. Copyright 2022, American Chemical Society. (**c**) Tetragonal rod-like nanomaterials interconnected with carbon nanofiber surfaces used as super-capacitor electrodes exhibited high specified capacitance [186]. Copyright 2023, American Chemical Society.

Supercapacitors could improve performance by varying the morphology of electrospun nanofibers. In particular, porous carbon nanofibers are actively studied to maximize the supercapacitor performance due to their high surface-to-volume ratio with the electrolyte, thereby efficiently transporting electrons in the longitudinal direction. For instance, Nam et al. electrospun the solutions using mixed PAN and PS to obtain polymer nanofibers (Figure 19a) [184]. During carbonization, PS is used as a sacrificial substance to generate pores inside the multiporous carbon nanofibers. The carbon material has a high-capacity value of 202.4 F·g^−1^ at a current density of 1 A·g^−1^. Moreover, to improve the capacitance performance, introducing heterogeneous elements to the CNF’s surface by oxygen plasma treatment was conducted. As a result, the capacity value is enhanced more than 1.5 times the non-plasma-treated CNFs (358.2 F·g^−1^ at 1 A·g^−1^). In another approach, Serrano et al. developed a strategy to generate flexible porous CNFs derived from electrospun PMMA-block-PAN copolymers nanofibers (Figure 19b) [185]. The porous carbon fibers (PCFs) have different shapes depending on humidity due to the vapor-induced phase separation. Therefore, electrical double-layer capacitance and flexibility were effectively achieved at an intermediate range of 70% RH, showing 249 F·g^−1^ capacitance value and 97.3% cycling stability. In an effort to further improve the supercapacitor performance, Acharya et al. are attracted by the moderated thermal transformation ability of metal−organic frameworks (MOFs), which empowers the synthesis of nanomaterials with precisely controlled porosities and morphologies (Figure 19c) [186]. Here, iron MOF and MIL-88A were used as the source of Fe_2_O_3_/nanoporous carbon (NPC) and Fe_3_C, respectively. Through electrospinning using a mixed solution of MIL-88A, PMMA, and PAN, following hydrothermal treatment and carbonization to obtain Fe_2_O_3_/NPC decorated on Fe_3_C implanted porous carbon nanofibers. The resulting MOF-derived electrode materials exhibit a high specific capacitance of 531 F·g^−1^ at 1 A·g^−1^. It is worth noting that porosity plays an important role in enhancing supercapacitor performance, and highly porous supercapacitor electrode materials can simply be obtained using an electrospinning system.

### 3.4. Battery Electrode

**Figure 20:**
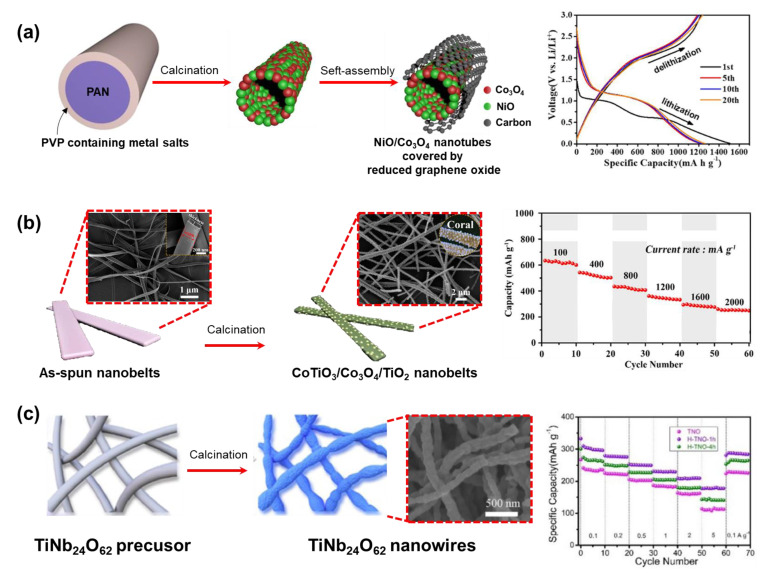
(**a**) High-rate capacity and cycle stability of anode made from NiO/Co_3_O_4_ nanotubes encapsulated with reduced graphene oxide wall [193]. Copyright 2020, American Chemical Society. (**b**) The unique coral-like CoTiO_3_/Co_3_O_4_/TiO_2_ hybrid nanobelts structures anode benefits the lithium ions transformation and provides excellent support for the specific capacity [194]. Copyright 2019, Wiley. (**c**) TiNb_24_O_62_ porous nanowires have lots of oxygen vacancies providing more pseudo-capacity behavior activity for the anode [198]. Copyright 2021, Elsevier.

Since the early years of the 21st century, researchers have believed that to improve the capacity of lithium batteries, the graphite anode should be replaced by another anode material that has a relatively small molecular weight, low density, a favorable stoichiometric ratio for accepting Li, and higher retention ability during repeated charge–discharge cycles [187]. Metal oxide (MO) has emerged as an ideal material to replace graphite because of both its superior capacity and its low cost and facile synthesis [188,189]. However, the naturally poor electrical conductivity and resistance to pulverization during lithiation/delithiation remain challenges. To address these issues, experts approached the electrospinning technique to fabricate metal oxide nanofibers [190]. Cobalt oxide (Co_3_O_4_) and nickel oxide (NiO) nanofibers were obtained as alternative anode materials through a similar process [191,192]. However, their capacity and retention are still insufficient to meet the demand for large-scale applications. The combination of metal oxides opened a new orientation towards improving anode materials due to the abundant electrochemical activities, more redox reactions, and especially the synergistic effect between two different metals. For example, Dai et al. designed an anode for lithium-ion batteries with NiO/Co_3_O_4_ nanotubes encapsulated with reduced graphene oxide using an electrospinning technique (Figure 20a) [193]. As a result, the anode showed large lithium-ion storage capability (~1206 mAh·g^−1^ at 0.1 A·g^−1^ after 100 cycles), and high-rate capacity and cycle stability. Here, the reversible reaction of the lithium-ion with NiO and Co_3_O_4_ (conversion-type capacity), the pore of the tubular structure, and the surface of reduced graphene oxide (adsorption-type capacity) all contribute to the specific capacity. Another interesting case is Liu et al.’s fabrication of lithium-ion batteries anode from one-dimension hierarchical coral-like CoTiO_3_/Co_3_O_4_/TiO_2_ hybrid nanobelts via a two-step electrospinning calcination strategy (Figure 20b) [194]. This electrode material offers a combination of advantages from metal components (CoTiO_3_ (enhancing cyclic stability), Co_3_O_4_ (high theoretical specific capacity), and TiO_2_ (improvement of structural stability)), and all advantages are reflected in the electrochemical performance (capacity of 722.3 mAh·g^−1^ at 100 mA·g^−1^ after 250 cycles). Moreover, the unique coral-like nanobelt structures benefit the lithium ions transformation, and then provide excellent support for the specific capacity.

One group that cannot be ignored in the family of alternative anode materials is the intercalation-type metal oxide materials (e.g., Li_4_Ti_5_O_12_, CuFe_2_O_4_, ZnCo_2_O_4_, and TiNb_24_O_62_) which are well-known for their low-toxicity, reduction total cost, and tunability of working voltage and energy density by varying the metal ratio [195,196,197]. However, like other metal oxide groups, this material must have a tailored design in the nanostructures to shorten the dispersion distances of lithium ions and enhance the specific capacity. For example, instead of using solvent–thermal or template methods (which are complex and require additional templates) to generate TiNb_24_O_62_ porous nanoparticles, Zhu et al. used simple electrospinning with a subsequent hydrogenation method to create the material in the form of a one-dimensional nanowire with lots of oxygen vacancies (Figure 20c) [198]. As expected, the designed material exhibits a faster lithium-ion diffusion path and more pseudo-capacity behavior activity than the bulk material. The electrospinning technique can be seen to have played a significant role in enriching the structural morphology and improving the electrochemical performance of lithium-ion batteries.

### 3.5. Chemical Sensor

**Figure 21:**
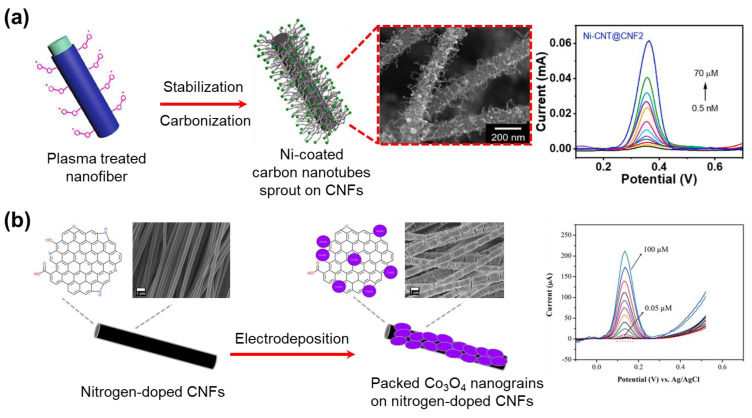
(**a**) Nickel-decorated multidimensional CNFs applied in acetaminophen detection [200]. Copyright 2023, Elsevier. (**b**) Cobalt oxide nano-grains on nitrogen-doped CNFs applied in dopamine detection [201]. Copyright 2022, Elsevier.

Electrochemical sensors enable the export of electrical signals produced by interactions between the analytes [199]. The material of electrochemical sensors could be obtained by attaching metal or metal oxide to the surface of CNFs. Phan et al. report a strategy to generate nickel-decorated multidimensional CNFs via electrospinning combined with oxygen plasma treatment and carbonization (Figure 21a) [200]. Here, nickel particle-coated carbon nanotubes sprout on the surface of the CNFs through the catalytic reaction of nickel and decomposed PVP during the carbonization. This creates a large active surface area supporting rapid electron transfer leading to effective electrochemical detection. As a result, this CNFs-based electrochemical sensor has a high sensitivity (25.29 μA·μM^−1^ cm^−2^ for 0.027 cm^2^ active surface area) and a low limit detection (0.5 nM) to acetaminophen molecules. Based on facile electrospinning and electrodeposition, Yin et al. fabricated packed cobalt oxide nanograins on nitrogen-doped CNFs to assemble a dopamine electrochemical sensor (Figure 21b) [201]. The high surface area of the carbon nanofiber matrix and the homogeneous dispersion of Co_3_O_4_ led to effective dopamine detection. In addition, the carbon skeleton supports electron transfer within the electrode surfaces. This sensor showed a low limit detection (9 nM) over a wide range of concentrations of (0.01 to 100) µM, superior sensitivity, and excellent selectivity for dopamine detection.

**Figure 22:**
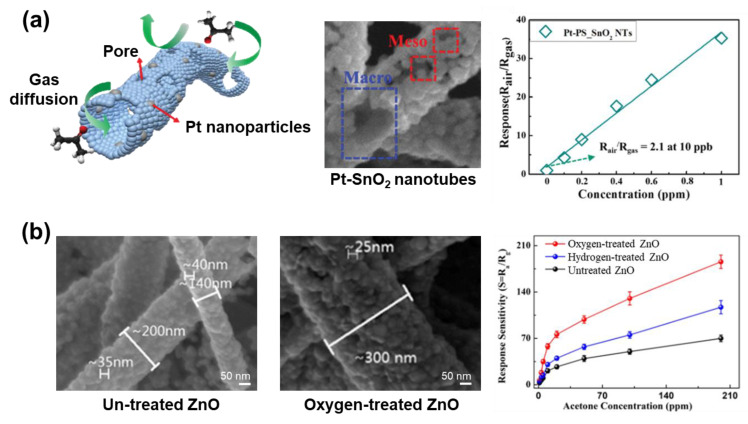
(**a**) Pt acts as a catalyst contributing to enhanced selectivity and sensitivity of Pt porous SnO_2_ nanotubes [203]. Copyright 2016, Wiley. (**b**) Oxygen-plasma-treated ZnO nanofibers show excellent acetone-sensing properties over the untreated ones [204]. Copyright 2020, American Chemical Society.

In recent years, attention to electrospinning metal oxide nanofibers in gas sensing applications has increased due to their unique structures and good electrical properties [202]. Needless to say, the unique morphology facilitates the efficient penetration of the target gas into the active metal oxide layer, which is the main reason for the high sensitivity of metal oxide gas sensors. Another approach to enhance sensor performance is to use noble metals to decorate metal oxide nanofibers. Using electrospinning combined with the calcination process, Jang et al. easily decorated Pt homogenous catalyst carriers on porous SnO_2_ nanotubes (Figure 22a) [203]. Here, PS was added into the electrospun solution as a sacrificial template to generate meso- and macro-pores on material structure. Moreover, noble metals dispersed in the material work as effective catalysts by decreasing the activation energy of gas chemisorption. These two metrics create a synergistic effect to significantly improve the selectivity and sensitivity of the sensors. In detail, the sensor showed superior selectivity against other interfering gases (including H_2_S, toluene, pentane, CO, NO, NH_3_, CH_4_, and H_2_) and the capability of detecting low-level acetone (10 ppb). To induce a change in the surface of nanofibers, as well as the addition of a hetero-component to the as-spun solution, exposing the nanofiber to oxygen plasma also promotes an increase in the sensor’s response. For example, oxygen-plasma-treated ZnO nanofibers show excellent acetone-sensing properties, due to their specific surface area and porosity increase (Figure 22b) [204]. After plasma treatment, more pores and a larger specific surface area were achieved on ZnO nanofibers. Moreover, the bandgap of oxygen-exposed ZnO nanofibers significantly changed to near Fermi level, leading to charge transfer and further expansion of the charge depletion region [205]. As a result, ZnO nanofibers exposed to plasma oxygen gas exhibited an approximately two-fold increase in response sensitivity, compared to untreated nanofibers.

## 4. Conclusions

Electrospinning is a method of manufacturing nanofibers. Though the materials for the electrospinning processes are usually polymer solutions, the treatments on the nanofibers and extensive choices of materials allow many kinds of materials to be produced. To date, the materials that can be provided by electrospinning include polymer, polymer composite, metal, and metal oxide nanofibers. On the other hand, modifications to the electrospinning system configuration can lead to various changes in the collected nanofiber membrane. Firstly, not only does the use of special nozzles and collectors alter the morphology of the electrospun nanofibers, but the combination of different solvents and materials in one single electrospun solution can also lead to the same results. Furthermore, the after-treatment carried out on the nanofibers can convert them into different materials. Especially carbon nanofibers, which have a significantly high surface area created from the removal of sacrificing materials, can be harvested from the carbonization of electrospun nanofibers. Secondly, the addition of extra parts, which are used to control the pathway of the electrical field, can align the nanofibers following a designed pattern. In short, there is no limitation in the morphology and alignment of nanofibers that can be produced by electrospinning, opening up the potential to be further developed in the future. However, the disadvantages of the electrospinning process are that it is highly dependent on the environment humidity and stability of the power source, restricting the use of the technique in specific conditions. The applications of these nanofibers were also introduced in the review. It is clear that the electrospun nanofibers have been used in many applications, from simple applications, such as sound absorption and air purification, to highly advanced applications, like sensors, catalysts, and energy storage. The electrospinning method has great potential to provide materials with excellent properties, while it also offers many opportunities for further improvement.

## Figures and Tables

**Table 1:** Simple polymer solutions that had been used in electrospinning.

Polymer	Solvent	Reference
PEO ^1^	Water/chloroform	[37]
PVA	Water	
CA	Acetone	
PANi ^2^/PEO	Chloroform	[38]
PEO	Isopropyl alcohol	[39]
Polycarbonate	DMF ^3^/THF ^4^	
Polyurethane	DMF	[40]
Polycaprolactone	Acetone	[41]
PVP	Ethanol/water	[42]
PANi	Formic acid	[43]
Ppy ^5^	DMF	[44]
Magnesium linked PEDOT:PSS ^6^	DI water ^7^	[45]
Collagen	HFIP ^8^	[46]
Gelatin	Aqueous acetic acid (90%)	[47]
HA-DTPH ^9^	Dulbecco’s modified eagle’s medium	[48]

^1^ Polyethylene oxide. ^2^ Polyaniline. ^3^ N,N-dimethylformamide. ^4^ Tetrahydrofuran. ^5^ Polypyrrole. ^6^ poly(3,4-ethylenedioxythiophene) polystyrene sulfonate. ^7^ De-ionized water. ^8^ 1,1,1,2,2,2-hexafluoro-2-propanol. ^9^ 3,3′-dithiobis(propanoic dihydrazide)-modified hyaluronic acid.

**Table 4:** The solutions for the electrospinning of polymer composite nanofibers.

Polymer Carrier	Solvent	Filler	Reference
PVA	DI water	Ag NPs	[84]
Polyvinyl butyral	Isopropyl alcohol/water	Fe(NO_3_)_3_, Co(NO_3_)_2_, or Ni(NO_3_)_2_	[85]
PVA	DI water	Cu(CH_3_COO)_2_	[86]
PVP	DMF/DI water	H_2_PtCl_6_	[87]
PVP	DMF/DI water	H_2_PtCl_6_/HAuCl_4_	
Poly(acrylic acid)	Ethanol	HAuCl_4_	[88]
PVP	Ethanol	Al(CH_3_COCHCOCH_3_)_3_	[89]
PVA	DI water	Ce(NO_3_)_3_	[90]
PMMA	DMF/chloroform	C_4_H_6_MnO_4_	[91]
PVA	DI water/propanol/and isopropanol	SnCl_4_	[92]
PVP	Ethanol/acetic acid	Ti(OBu)_4_	[93]
PAN	DMF	Melamine–trithiocyanuric acid	[94]

## Data Availability

Not applicable.
